# Characterisation of the rumen resistome in Spanish dairy cattle

**DOI:** 10.1186/s42523-021-00125-0

**Published:** 2021-09-22

**Authors:** Adrián López-Catalina, Raquel Atxaerandio, Aser García-Rodríguez, Idoia Goiri, Mónica Gutierrez-Rivas, José Antonio Jiménez‐Montero, Oscar González-Recio

**Affiliations:** 1 Departamento de Mejora Genética Animal, Instituto Nacional de Investigación y Tecnología Agraria y Alimentaria (INIA-CSIC), Crta. de la Coruña km 7.5, 28040 Madrid, Spain; 2 Department of Animal Production, Neiker-Basque Institute for Agricultural Research and Development, Basque Research and Technology Alliance (BRTA), Campus Agroalimentario de Arkaute, 01192 Arkaute, Spain; 3 Confederación de Asociaciones de Frisona Española, 28340 Valdemoro, Madrid, Spain; 4 Departamento de Producción Agraria, Escuela Técnica Superior de Ingeniería Agronómica, Alimentaria Y de Biosistemas, Universidad Politécnica de Madrid, Ciudad Universitaria s/n, 28040 Madrid, Spain

**Keywords:** Dairy cattle, Ruminal resistome, Antibiotic resistance, Ruminal microbiota, Antimicrobial resistance genes, Metagenomics, Nanopore, MinION, Long-reads

## Abstract

**Background:**

Rumen microorganisms carry antimicrobial resistance genes which pose a threaten to animals and humans in a One Health context. In order to tackle the emergence of antimicrobial resistance it is vital to understand how they appear, their relationship with the host, how they behave as a whole in the ruminal ecosystem or how they spread to the environment or humans. We sequenced ruminal samples from 416 Holstein dairy cows in 14 Spanish farms using nanopore technology, to uncover the presence of resistance genes and their potential effect on human, animal and environmental health.

**Results:**

We found 998 antimicrobial resistance genes (ARGs) in the cow rumen and studied the 25 most prevalent genes in the 14 dairy cattle farms. The most abundant ARGs were related to the use of antibiotics to treat mastitis, metritis and lameness, the most common diseases in dairy cattle. The relative abundance (RA) of bacteriophages was positively correlated to the ARGs RA.

The heritability of the RA of the more abundant ARGs ranged between 0.10 (*mupA*) and 0.49 (*tetW*), similar to the heritability of the RA of microbes that carried those ARGs. Even though these genes are carried by the microorganisms, the host is partially controlling their RA by having a more suitable rumen pH, folds, or other physiological traits that promote the growth of those microorganisms.

**Conclusions:**

We were able to determine the most prevalent ARGs (*macB, msbA, parY, rpoB2, tetQ* and *TaeA)* in the ruminal bacteria ecosystem. The rumen is a reservoir of ARGs, and strategies to reduce the ARG load from livestock must be pursued.

## Background

The rumen resistome is the compound of all the antimicrobial resistance genes (ARGs) carried by the microbes that inhabit the rumen. These microorganisms create a very complex ecosystem made up of bacteria, archaea, viruses, fungi and protists, among others. Studying their relationship, their roles in the process of digestion and how they transmit ARGs can help to understand the current state of the antimicrobial resistance (AMR) in livestock. The World Health Organization (WHO) warned about the issue with antimicrobial resistant pathogens, as it is predicted that by 2050 multi-resistant bacteria will kill 10 million people per year, surpassing cancer as our main health concern. Among the 1,461 diseases recognised in humans, 60% of them are caused by multi-host pathogens capable of moving across species. Moreover, roughly 75% of the newly detected infective diseases over the last 30 years have been zoonotic [1]. Here lies the importance of characterizing the rumen resistome, as ARGs could jump from faeces and saliva within and across species, arriving to humans via direct contact, through the food chain or disseminated in the environment (e.g. manure). A good approach to reduce the risks of the emergence of AMR is to understand how they appear, their relationship with the host, how they interact or how they are transmitted to humans, animals and the environment. This is the main goal of the One Health Initiative, which is the integration of human, animal and environmental health under the same framework.

The most remarkable role of modern medicine is being able to prevent and cure life-threatening infectious diseases, which is becoming a problem as AMR pathogens are gaining prevalence. Bacteria are gaining ARGs, becoming resistant or multi-resistant to diverse drugs such as cephalosporins, quinolones and penicillases, among others [2]. In livestock, the acquisition of resistance to antimicrobials is still not well understood but may be largely promoted by the over and misuse of antibiotics (ATBs) to treat or prevent diseases or as growth promoters. The use of ATB with prophylactic aims was forbidden in Europe to avoid the presence of ATB residues in the milk [3]. Using antimicrobial feed additives on livestock as growth promoters was prohibited in Europe in 2006, but it is still a problem in other countries as it increases the abundance of ARGs in the gut and faeces [4]. Mastitis is the most common and costly disease affecting dairy cattle and it is responsible for the majority of antibiotic use [5]. Mastitis is an intramammary infection (IMI) caused mostly by staphylococci, streptococci and Gram-negative bacteria although more than 135 microorganisms can cause this disease [6]. Mastitis treatment and dry cow therapy account a significant proportion of the total antimicrobial usage. A large amount of ARGs related to this disease is expected to be found as most of the antibiotic used in dairy cattle are related to it.

Under the One-Health approach, dissemination of AMR from livestock can become a problem due to the use of livestock faeces and urine excretions, most of these contaminated with milk residues disposed of in the slurry pond and excreta from antibiotic-treated animals, as manure and organic fertiliser. It is important to understand that bacteria have mobile genetic elements (MGE) such as transposons and plasmids as well as horizontal gene transfer (HGT) or bacteriophages to pass genetic material to other species. The ARGs could jump from faeces to soil bacteria, then to plants, and then to animal fed in a circular manner [7]. This is turning into an important issue to address because antibiotic-resistant microbes detected are becoming a threat to human health in the same way as the resistances originating at human levels will be a threat for the animal and environmental health. It represents a problem to livestock industry not only for the inherent risk for both animal and human health but also because of the increase of infectious disease morbidity and mortality coming together with the appearance of new opportunistic microorganisms with ARGs. This is an added cost to the industry as new remedies and prevention measures will need to be developed [8].

Regarding the health risk mentioned above, there are two possible ways for AMR to affect humans. The first one will be direct exposure to antimicrobial-resistant bacteria by contacting with host animals (livestock and pets) [9] and the second one is via the food chain or contamination of the meat and milk by commensal and pathogenic AMRs microbes [10].

Strategy is to modulate the composition of the ruminal microbiota must be considered. Diet or the environment determine the microbiota composition, but there is also a host effect regulating its composition [11]. This information can be implemented into animal breeding programs to select animals with a microorganism community less susceptible to carry or transfer ARGs, as well as animals with fewer pathogens and opportunistic microorganisms in the rumen.

The objectives of this study were (1) to identify the antimicrobial resistance genes present in the dairy cattle rumen and describe their composition and (2) determine the host genetic effect on the relative abundance of ARGs in order to understand the role of selective breeding to modulate the presence and dissemination of ARGs.

## Results

### Relative abundance of antimicrobial resistance genes by herd and resistance family

**Fig. 1:**
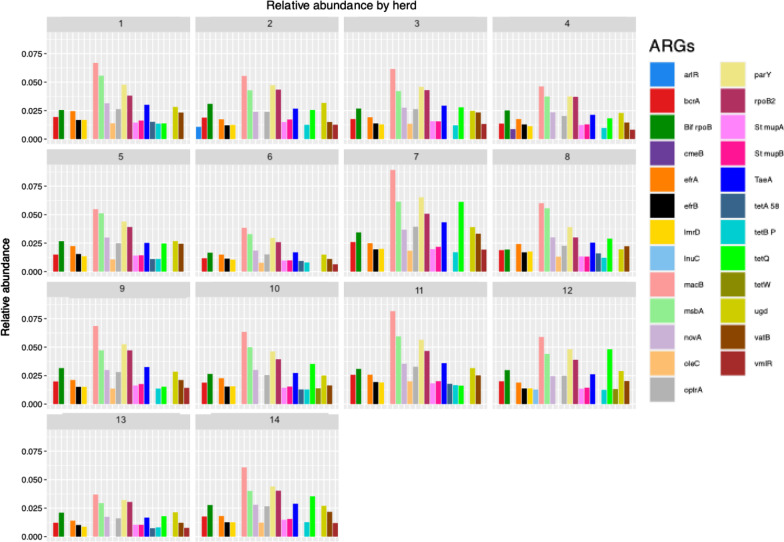
Relative abundance (%) of ARGs by herd. Each box represents a different farm while each bar is the RA of a given gene

A total of 998 genes were detected in the ruminal microbiome. Sixty-nine AMR genes with a prevalence larger than 0.005% in all the herds were used in downstream analyses. The relative abundances (RA) of the 25 most abundant in each herd were plotted (Fig. 1) showing consistency among farms. The high prevalence of several tetracycline (TE) resistance genes (*tetA(58), tetB(P), tetW, tetQ)* is not surprising as it is one of the most common antibiotics used in a wide range of applications to treat many different infectious diseases, not only in the field of bovine medicine.

The most prevalent AMR genes were *macB, masbA, parY, proB2, tetQ* and *TaeA*, (Table 1). The *macB* gene, which confers resistance to macrolides, presented the largest relative abundance (around 0.05%) in all herds.

**Table 1:** The 6 most prevalent antimicrobial resistance genes found in rumen and the characteristic of the antimicrobials to which they confer resistance

Gene	Antibiotic class	Mechanism	Disease
*macB*	Macrolide	Efflux pump	Mastitis [12] Metritis postpartum [13]
*msbA*	Nitroimidazole	Efflux pump	Genital trichomoniasis in cattle Main growth-promoter [14]
*parY*	Aminocoumarin	Target alteration	Mastitis in dry dairy cattle [15]
*rpoB2*	Rifamycin	Target alteration and protection	Mastitis [16]
*tetQ*	Tetracycline	Target protection	Post-partum metritis Lameness and mastitis [15]
*TaeA*	Pleuromutilin	Efflux pump	Mycoplasma in swine [12]

**Fig. 2:**
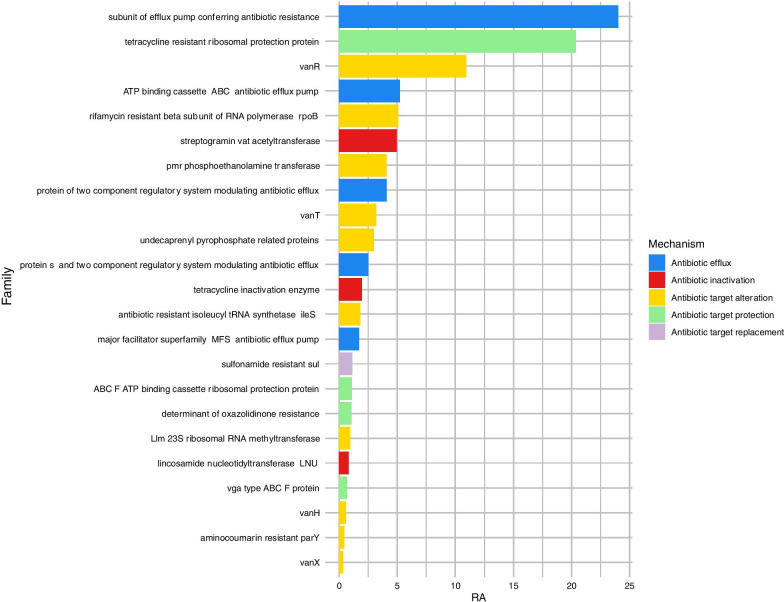
Relative abundance (%) of the antimicrobial resistance families. The colour represents the resistance mechanisms associated with each family of AMR

Genes where also grouped according to the family of resistance to which they belong to (Fig. 2). The most abundant family was a subunit of efflux pump conferring antibiotic resistance, with around 25% of the ARGs abundance belonging to it. The second one was the tetracycline resistance proteins, grouping around 20% of the genes. The third most abundant family was the VanR family with around 13% of the total ARG abundance.

### Relative abundance of resistances according to the antibiotic category

**Fig. 3:**
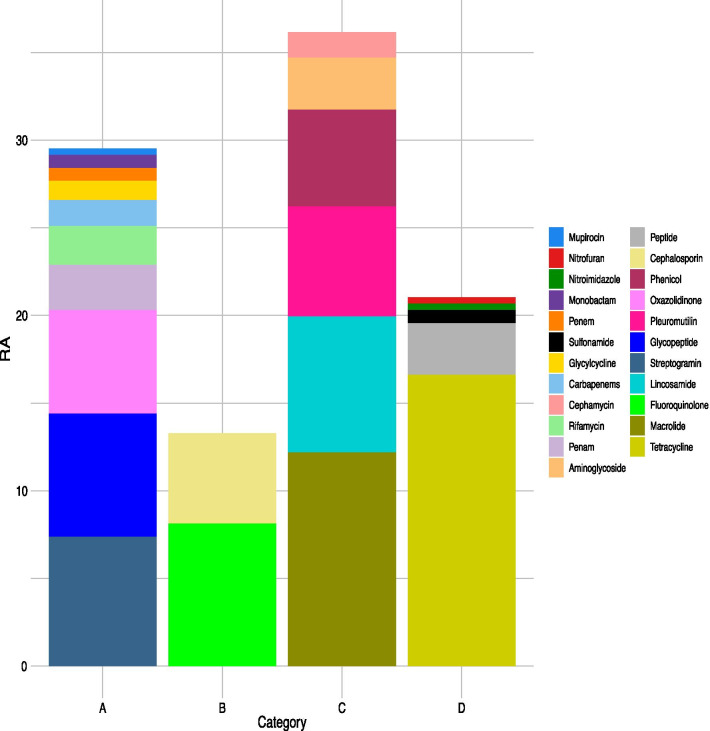
Relative abundance (up to 100%) of the genes with a prevalence larger than 0.005% classified according to the categorisation of antibiotics in the European Union for use in animals to which they confer resistance

Genes were also classified according to the categorisation of antibiotics made by the European Medicines Agency (EMA) [17] (Fig. 3). A total of 57% of the genes belong to category C and D, being tetracyclines, macrolides and lincosamides the most representative. Forty-three percent of them were grouped under animal-restricted categories B and A, being of special concern fluoroquinolones, cephalosporines (3rd - and 4th- generation), glycopeptides and streptogramins, as they belong to restricted categories according to the EMA.

This categorisation is based on when the antibiotic should be used. Antibiotics of category A should be avoided in veterinary medicine. Those of category B are critical to human medicine and its use is restricted in livestock and very limited in pets. Category C groups those antibiotics to which we have alternatives in veterinary medicine and Category D gathers the first-choice antibiotics in veterinary medicine.

### Bacteriophages

**Fig. 4:**
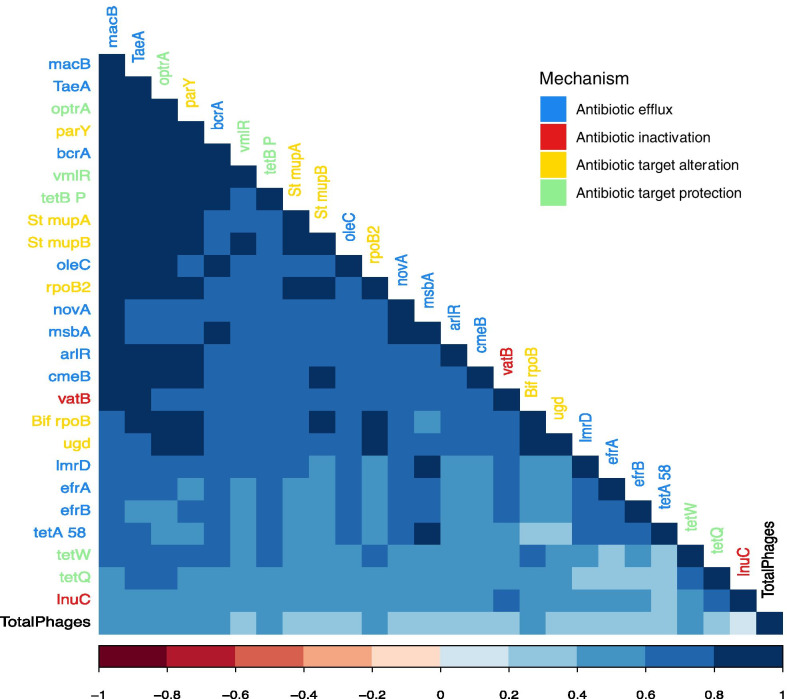
Phenotypic correlation of antimicrobial resistance genes with bacteriophages. Colours in the names of the genes refer to the mechanism of resistance

The association between the abundance of ARGs and bacteriophages was studied, as the latter play a role in ARG transfer between bacteria. Figure 4 shows that the phenotypic correlation between the bacteriophages and the genes were positive and > 0.20 (*P* value < 0.05). It was also observed that most of the ARGs had a large and positive pairwise correlation (0.4–0.8). A cluster of several ARGs (*macB, TaeA, optrA, parY, bcrA*, *TetBP, vmlR, rpoB2, Bif rpoB* y *tetW*) was observed with pairwise correlations larger than 0.6 for their relative abundances.

### ARGs heritability

**Fig. 5:**
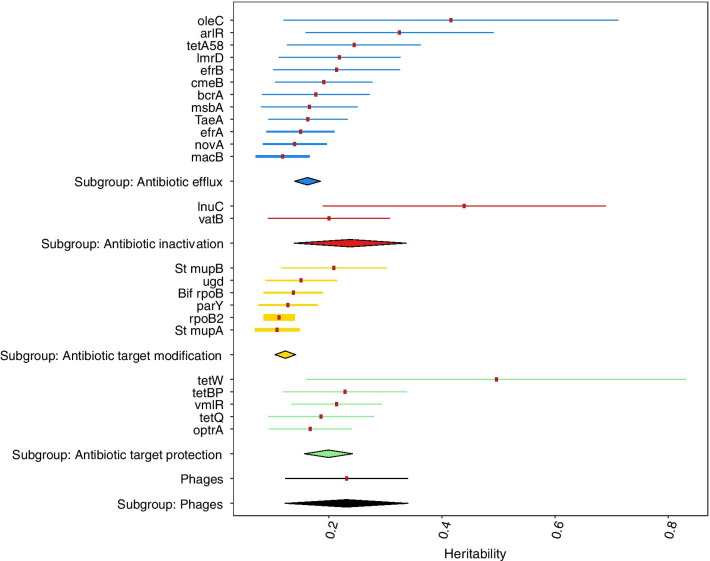
Heritability estimates for the relative abundance (RA) of the 25 most prevalent ARGs (by action mechanism) and the RA of total bacteriophages. The heritability values are represented by the red points and the HPD95 by the lines around them. The diamond represents the pooled result of that subgroup

The heritabilities for the RA of ARGs were estimated, ranging from 0.10 to 0.49 with a median of 0.18 and a mean of 0.21 (Fig. 5). The ARGs that showed a larger association with the abundance of bacteriophages (*macB, msbA, Bif rpoB, optrA, rpoB2, bcrA, parY, tetQ*) showed slightly lower heritabilities. Large posterior standard deviations were observed for the heritability estimates in all ARGs. The relative abundance of bacteriophages showed a heritability estimate of 0.21 (0.13–0.36).

The mechanism with the higher heritability estimate was the antibiotic inactivation (0.22) followed by the target protection (0.2), efflux pumps (0.18) and target modification (0.12).

### ARG-sharing networks

**Fig. 6:**
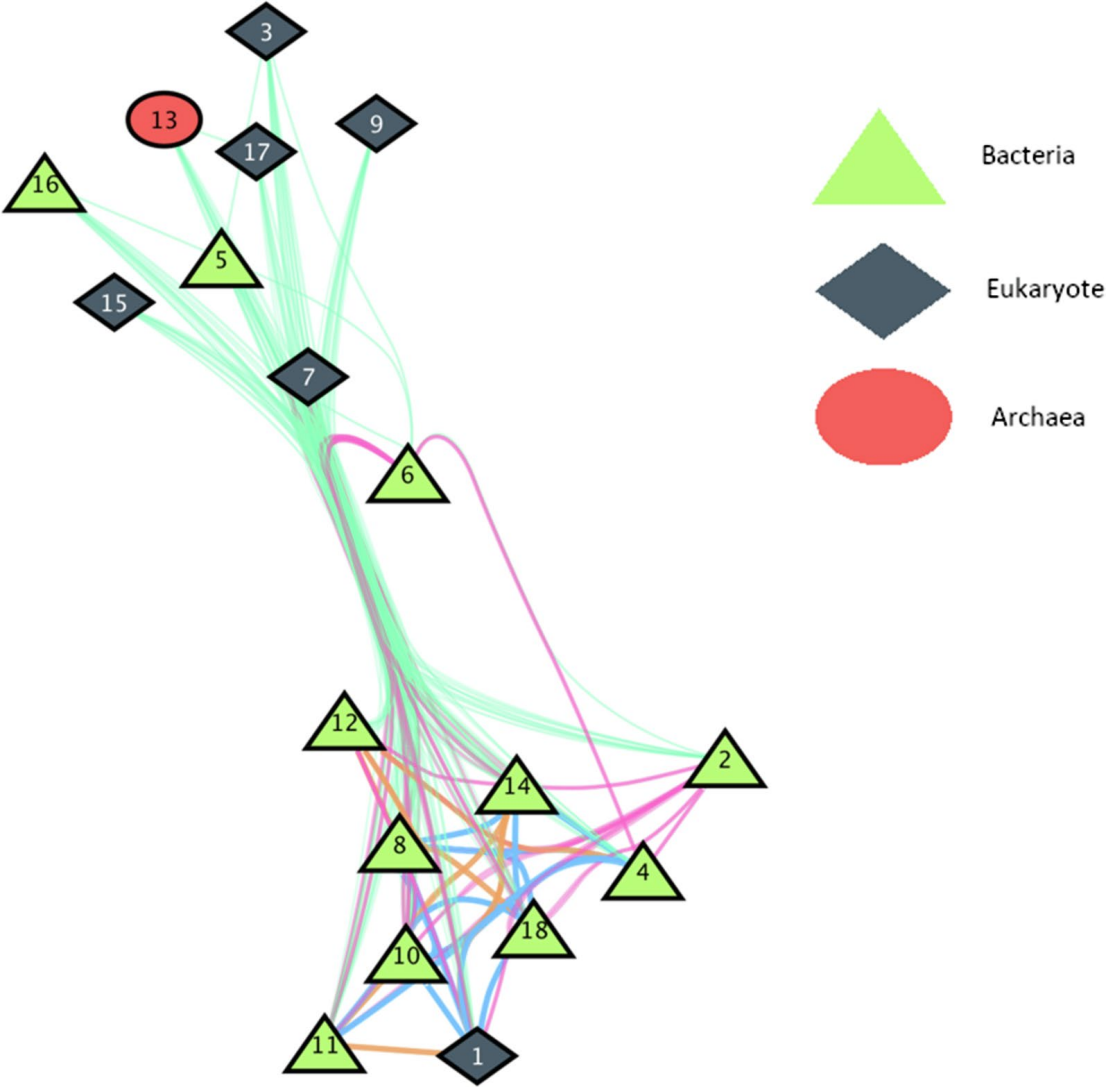
Dairy cattle ruminal ARG-sharing network. Nodes represent the taxonomic rank codified as shown in Table 2. The colour of the lines represents the strength of the relationship being 1, in light green, the weaker; 2 in pink and 3 in orange the medium, and 4 in blue the stronger. Two phyla or kingdom are connected if they share ARGs in all the farms analysed, having no coincidence in only one of them would discard the connection

We constructed a global ARG-sharing network including the 25 most prevalent genes in the 14 farms. Reads containing those genes were assigned to a taxonomical rank, which was possible provided that long reads were used. A total of 15 phyla in 3 superkingdoms shared these 25 genes (see Table 2 for the phyla and their codification). Fifty-five percent (55.28%) of the phyla carried between 2 and 7 of these ARGs. The remaining phyla shared even a larger number (> 7) of ARGs. Between 13 and 18 ARGs were shared by 6.83% of the phyla, and 13.67% shared > 19 ARGs. The central cluster mainly included protists, Actinobacteria, Bacteroidetes, Firmicutes, Proteobacteria, Spirochaetes, Tenericutes and Fibrobacteres, sharing most of the ARGs analysed between them (Fig. 6).

**Table 2:** Codification of the taxonomies for the sharing networks

Taxonomic class	Code	Taxonomic class	Code	Taxonomic class	Code
k_Eukaryota	1	p_Ciliophora	7	p_Euryarchaeota	13
p_Actinobacteria	2	p_Firmicutes	8	p_Fibrobacteres	14
p_Ascomycota	3	p_Mucoromycota	9	p_Basidiomycota	15
p_Bacteroidetes	4	p_Proteobacteria	10	p_Elusimicrobia	16
p_Cd Melainabacteria	5	p_Spirochaetes	11	p_Oomycetes no NCBI	17
p_Cd Saccharibacteria	6	p_Tenericutes	12	k_Bacteria	18

The cluster at the bottom was mainly made up by bacteria which share a large amount of ARGs. This can be explained by the common horizontal gene transfer processes that occurred between these microbes.

**Fig. 7:**
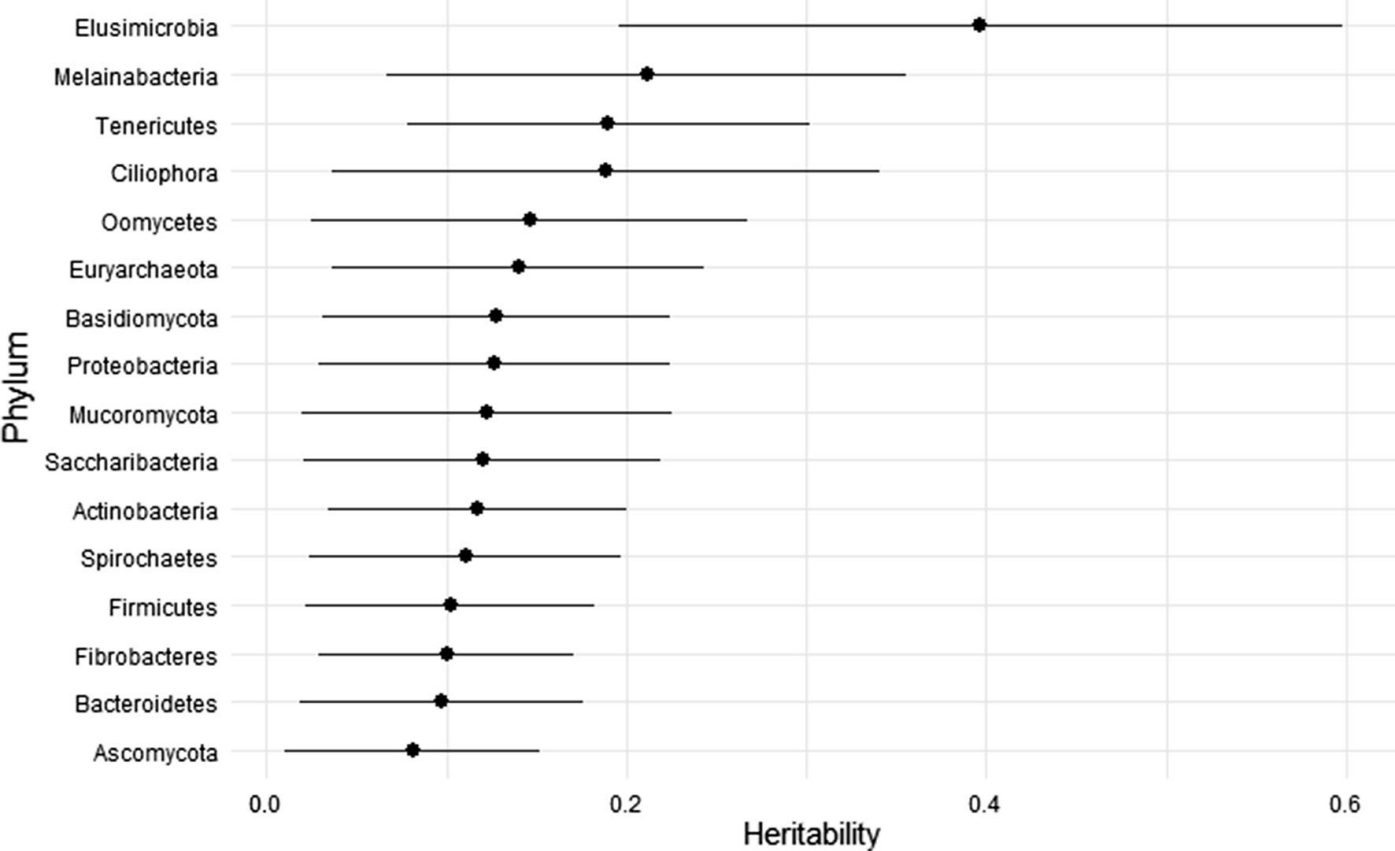
Heritability of the phylum that share the larger amount of ARGs. The plot shows the heritability of these phyla and its standard deviation

We estimated the heritability of the phyla RA in the ARG-sharing networks. The estimations ranged from 0.08 (± 0.06) for Ascomycota to 0.39 (± 0.20) for Elusimicrobiota. The average heritability of the RA of these microbes carrying ARGs was 0.15. A representation of these heritability estimates is provided (Fig. 7).

## Discussion

The effects associated to the rising of antimicrobial resistant microorganisms are currently of main concern. Most of the zoonotic processes are caused by pathogens that can affect both animals and humans, but antibiotics are frequently used to battle secondary infections in both human and animal health. For instance, during the COVID-19 pandemic, the WHO warned about how the misuse of antibiotics in COVID-19 mild cases could worsen the situation regarding AMR and encouraged to use them only under clear signs of bacterial infections [18]. The WHO has been advising since 2017 that veterinarians or practitioners should stop using antibiotics in healthy livestock animals to prevent the spread of antibiotic resistance. Thus, it is important to implement surveillance mechanisms to be aware of the ARGs reservoirs that can be present in livestock.

Not all the ARGs present in the rumen spread to the environment, as this depends on the shedding of the ARGs-carrying bacteria from the gastro intestinal tract which will then pass to the faeces, then to the soil if we use these as manure, then to plants and finally to the food chain. ARGs are rarely obtained by selective pressure of antibiotics. These genes are most likely obtained by HGT between environment bacteria [19] or by the transition of the animal from preruminant to ruminant, which is the key moment when the cow ruminal microbiota is first settled [20]. In order to get a deeper insight on how the AMR reach into the rumen, it is necessary to have all information on the antibiotic usage in each farm with individualized records in different stages of the animal life, mainly from its earliest stages, which is usually difficult to obtain under commercial conditions.

We found that the RAs of the most abundant ARGs was similar among farms, showing that the resistance to those ATBs are consolidated in the farms in northern Spain. Even though we found 998 ARGs making up to 1% of the total number of reads, only 69 of them had a RA > 0.005%, similar results have been shown in other studies [21].

The most abundant resistance family found was the efflux pump, which also happened in other studies [22]. Around 25% of the most prevalent ARGs belonged to this category in which the bacterium must use energy to eliminate the antibiotic. Processes and mechanisms mediated by ATP usually require a selective pressure to be maintained, as evolution tends to remove processes that consume energy if they are not important for the survival of the microorganism [23]. The second most abundant family was the tetracycline resistance proteins. This is not surprising as the use of tetracycline is of high importance in the most relevant diseases in dairy cattle and livestock (i.e. metritis and lameness) [15]. The third most abundant family of resistance was the one conferring resistance to vancomycin (van), a glycopeptide antibiotic with forbidden use in livestock. Resistance to this antibiotic is of main concern, as it is used as a last-line defence in life-threatening infections mediated by Gram-positive bacteria. Vancomycin is used against methicillin-resistant Staphylococci and Enterococci. If methicillin-resistant bacteria also gain resistance against vancomycin, there are very few alternatives to treat the infection. Staphylococci are already multi-resistance carriers, posing a serious threat to human and animal health. This family of AMR can be also explained by the use of avoparcin, an analogous of vancomycin as a feed additive in dairy cattle [24]. Resistance to some of these antibiotics have also been described in other studies such as Hui-Zeng Sun’s [25].

Macrolides, the antibiotic class associated to *macB,* are treatments of single administration to reduce the animal handling and facilitate its dosing [12]. The prevalence of *msbA* can be explained as nitroimidazoles were widely used as growth-promoters in the past decades, supporting the idea that these genes were not inherited independently in bacteria. We speculate that the resistance to an antibiotic that is no longer administered is only conserved if it there are other co-selection mechanisms present, as evolution tends to remove anything that causes an energetic cost if there is not a selective pressure on it [23]. When bacteriophages (mainly the caudovirales order) infect bacteria, virus-mediated HGT is likely to occur [26].

We classified the genes according to the categorisation of antibiotics made by the EMA for use in animals. Antibiotics belonging to category D should be first-choice treatment and those within category A must be restricted to last-line treatments in human health. Ideally, most of the ARGs detected in our samples should belong to Category C or D, because these categories include first-line treatments with alternatives in human medicine [17]. However, a large proportion of AMR from category A and B was observed in the rumen resistome. It must be emphasised that cephalosporins 3rd and 4th generation (category B) are used to treat respiratory infections, lameness and mastitis mainly due to the reduced withdrawal period in milk. Tetracyclines (category D) are a first-choice [15] treatment in a wide range of nature of dairy cattle and livestock diseases. Carbapenems (category A) are not used in cattle as their use is restricted to human health. The resistance to tetracyclines can be explained by two factors: tetracyclines are used via intrauterine to treat postpartum metritis as they are an effective and safe antibiotic with relatively low milk withdrawal period [13]. They also pose a risk in the environmental dissemination of ARGs due to discharges; the other one is the use of tetracyclines to treat some types of mastitis, one of the most common diseases in dairy cattle and the environmental contamination due to antibiotic residues in waste. The relatively large amount of resistance genes to antibiotics of the category A is worrisome as these antibiotics are strictly reserved to humans and companion animals under exceptional circumstances. Antibiotics are rarely given orally to dairy cattle. In most cases, they are administered by an intramammary or parenteral injection [13]. Many antibiotics are extracted from bacteria that are usually part of the environment. The resistance can be obtained as a normal response of a bacterium to the toxin of another one and not only as a response to the use of the antibiotic. This could be the case of carbapenems, which are last-resort antibiotic [27] used in human medicine but extracted from Enterococci species and *Escherichia coli*.

In this study, 43% of the analysed ARGs confer resistance to antibiotics of category A and B antibiotic, whereas 57% of them are included in category C and D. It must be pointed out that a bacterium could first develop resistance to a category D antibiotic, but the same gene may also confer resistance to antibiotics belonging to other categories due to chemical similarities between the molecules or the same mechanism of resistance in a process known as cross-resistance. This is the case of the identified *adeJ* gene, which confers resistance to carbapenem, rifamycin, diaminopyrimidine, tetracycline, phenicol, penem, macrolide, lincosamide, cephalosporin and fluoroquinolone. This gene might have been gained originally as a defence mechanism against tetracycline antibiotics (category D), but it also provides resistance to carbapenems. Furthermore, category C and D antibiotics are also usually administered topically, so these antibiotics might get into the rumen by contaminated feed or water or by licking the treated zone, although the topical application of medicine is not common in dairy cattle, limited mainly to wound care, and direct intramuscular injection is rather preferred [28]. The high correlations between ARGs suggest that there could be multi-resistant plasmids that usually carry those genes together. Most of these genes, that have also been described in other studies such as Ming-Yuan Xue’s [22], are involved in mechanisms of resistance of efflux pumps and target alteration. Among these, *macB, parY, rpoB2* and *TaeA* are related to the treatment of mastitis except *TaeA*. Although *msbA* is not present in this cluster, it was highly correlated with *macB*. This suggests that these ARGs are inherited or transmitted together. The high phenotypic correlation of the bacteriophages with the ARGs might open a new door to the use of these viruses to modulate the abundance of ARGs-carrying bacteria in the dairy cattle rumen. The current study shows that the role of the bacteriophages may be of interest as an indicator of ARG modulations, especially in early stages of the cow’s development. The RA of the bacteriophages partially depends on the genetic background of the animals, and the presence of the ARGs could increase in each generation of cows favouring HGT to happen.

Our results showed that the host genetics exert some control on the RA of ARGs, and bacteriophages, with low to moderate heritabilities.

The heritability of the RA of the ARGs needs to be interpreted as how the genetics of the cow control the variability of the relative abundance of these ARGs. There are certain physiological conditions (folds in the rumen, pH, feeding behavior, feed transit, etc.) that can promote the growth of certain microorganism strains that are related to a reservoir of ARGs. We observed that, even if these values are not as high as the ones for bacteriophages, they are good enough to be considered in future breeding programmes to reduce HGT and the amount of ARGs in the farms.

Besides, genetic correlation with other important traits must be estimated. The main phyla carrying these ARG also showed low to moderate heritability. The high heritability of phyla with a small presence in the ruminal ecosystem such as *Elusimicrobia* (h^2^ = 0.39)*,* whose role is not well understood yet [29], can be explained by a strong genetic effect of the host over this phylum. These phyla are not the most prevalent in the ruminal microbiota, as described in another analyses performed using the same data set [30]. However, they made up the core reservoir of ARGs in the rumen and it would be necessary to understand how these microorganisms with such a low RA interact with the environment and how they move across animals to disseminate ARGs.

Further studies are needed to understand why those genes with lower heritability also showed the higher correlations with the bacteriophages, and whether these are causal relationships.

The AMR core present in livestock and human population is one of the concerns for the One Health initiative. These resistances can jump to humans by indirect transmission from faeces used as manure for crops which could lead to the resistances passing to the plants and then to other animals or to humans. Direct transmissions through contact with the animals or with the intake of some of their products is also of concern. Reducing or, at least controlling, the antimicrobial resistances in livestock is also critical to human medicine.

## Conclusions

The results of this study showed that a core of ARGs was present in the rumen of dairy cattle. Most of the ARG were associated to prevalent diseases in dairy cattle such as metritis and lameness. Some of the detected ARGs belonged to category A and B and their presence in animal husbandry must be watched.

The heritability estimates for the RA of these ARG, and the microorganisms that carry them suggest that the host genotype partially determines the abundance of AMR. Bacteriophages showed an average heritability of 0.21, and were positively correlated with the RA of ARG. This is supported by the large relative abundance of Caudovirales, that make up almost the whole number of viruses found. The genetic parameters estimated in this work showed some potential for selection at modulating the presence of ARGs in the rumen microbiota.

Further studies are needed for a more in-depth characterisation of the resistome in the cow rumen, faeces and saliva as the main via to spread ARG. A better understanding of the resistome and it transfer into the environment are necessary to design more specific strategies towards the One Health concept. Selective breeding may be one of the option to reduce the circularity of ARG in the environment.

## Methods

### Data collection

Ruminal content samples from 14 commercial dairy farms in northern Spain were collected from 471 Holstein lactating cows. During sample collection, cows were placed in individual stalls and a tube (18 mm diameter and 160 cm long) was introduced down their oesophagus to their rumen. Around 100 ml were then pumped out (Vacuubrand ME 2SI, Wertheim, Germany) and stored in a container. Ruminal content was filtered using four layers of sterile cheesecloth and liquid fraction was immediately frozen in liquid nitrogen (N_2_) vapours. Frozen samples were transported to the laboratory in liquid N_2_ and stored at − 80ºC until analysed.

### Animal genotyping

Cows were genotyped using the EURO12K SNP chip from Illumina and imputed to 54,609 SNPs (Bovine 50 k SNP chip, Illumina) using BEAGLE software [31] and the Spanish reference population provided by CONAFE (Spanish Friesian Associations Confederation).

### DNA extraction and sequencing

Samples were thawed and homogenized using a blender before being analysed using the commercial kit “DNeasy PowerSoil” (Qiagen, Valencia, CA, USA). The concentration and purity of each sample were estimated using a NanoDrop UV/Vis Spectrophotometer (NanoDrop Technologies Inc.). The sequencing was performed using Nanopore Technology and the MinION sequencer, following the protocol from Oxford Nanopore Technologies (Oxford, UK) using the ligation sequencing kit (SQK-LSK109) and multiplexing 12 samples per run with the native barcoding kit (EXP-NBD104 or EXP-NBD114). Long-reads were obtained and processed as explained below. After quality control, a total of 416 were kept for downstream analysis. The data set included a total of 88,703,984 reads. The whole rumen content was sequenced, but cow, plant and phyla described in Table 3 genes were removed from the results.

**Table 3:** Phyla removed from the taxonomy

Acanthocephala	Chordata	Mollusca	Platyhelminthes
Annelida	Cnidaria	Nematoda	Porifera
Arthropoda	Ctenophora	Nematomorpha	Rhodophyta
Brachiopoda	Echinodermata	Nemertea	Rotifera
Bryozoa	Entoprocta	Onychophora	Streptophyta
Chaetognatha	Gnathostomulida	Orthonectida	Tardigrada
Chlorophyta	Hemichordata	Placozoa	Xanecoelomorpha

### Bioinformatic analyses

The base-calling was performed using the Guppy (v. 4.2.2) software by Oxford Nanopore Technologies with filter of quality score of QS > 7 and read length > 150 bp. After that, the sequences were analysed using the SQM_reads tool from SqueezeMeta [32]. This pipeline aligns each read to a gene reference database and provides the number of copies of each gene present in the sample. The Comprehensive Antibiotic Resistance Database (CARD v3.0.4) [33] was used to assign gene ontology for taxonomy and functional annotation. The pipeline was implemented in the CESGA super-computing centre in Galicia, Spain. Genes with a prevalence greater than 0.005% kept for downstream analyses, resulting in a list of 69 genes for which AMR gene family, class, resistance mechanism and microbiota composition were obtained.

### Compositional data

Read count from metagenome are compositional data (CoDa), as they are discrete vectors representing the numbers of outcomes falling into any several mutually exclusive categories.

Dealing with CoDa needs to differentiate between real and false zeros. For that, generalised Bayesian-multiplicative (GBM) replacement was used [34]. This technique is preferred when the total sum of a vector is uninformative, as in this case when the interest lays in the relative abundance of each gene. Let $c_{i} = \left( {c_{i1} , \ldots , c_{iD} } \right)$ be a compositional vector of counts, gene reads in this case. A zero was replaced by its posterior Bayesian estimate $E\left[ {\pi_{i} |c} \right] = \frac{{c_{i} + s \cdot t_{i} }}{n + s}$ using the following formula

$$r_{ij} = \left\{ {\begin{array}{*{20}l} {t_{ij} \cdot \le \frac{{s_{i} }}{{n_{i} + s_{i} }},} \hfill & { if\;x_{ij} = 0,} \hfill \\ {x_{ij} \cdot \left( {1 - \mathop \sum \limits_{{k|x_{i} k = 0 }} t_{ik} \cdot \frac{{s_{i} }}{{n_{i} + s_{i} }}} \right),} \hfill & { if\;x_{ij} > 0,} \hfill \\ \end{array} } \right.$$

Being $t_{ij}$ related to the prior, $s_{i}$ to its strength, $n_{i} = \mathop \sum \limits_{i} c_{ij}$ with $t_{j} = \frac{1}{D}$. The parameters may vary along the samples according to the information of the trials. The advantage of this technique is the preservation of the ratios between parts and the sum of the vector:

$$\frac{{r_{ij} }}{{r_{ik} }} = \frac{{x_{ij} }}{{x_{ik} }}; \mathop \sum \limits_{j = 1}^{D} r_{ij} = 1$$

Genes with a total sum of reads smaller than 3 were removed from the data set. Then, imputation of zeroes was implemented with the Geometric Bayesian multiplicative (GBM) method from the cmultRepl function of the zCompositions package in R [35].

### Variance component estimation

The heritability of the 25 most prevalent ARGs and the phyla that carry them were calculated using the software Threshold Model [36] including the genomic relationship matrix instead of the pedigree numerator matrix. The statistical model can be represented in algebraic notation as:

$$y_{RA\,ijkl} = \mu + LC_{j} + Herd_{k} + DIM_{l} + u_{i} + e_{ijkl}$$

where $y_{RA}$ was the relative abundance of the ARG or the phylum; $\mu$ was a population mean; $LC_{j}$ was lactation number; $Herd_{k}$ was the herd-month effect; $DIM_{l}$ was days in milk, categorized in three stages (0 to 60; 60 to 150; > 150 days post partum); $u_{i}$ was the additive genomic effect assumed distributed as ${\mathbf{u}}$ ~ N(0,**G**σ^2^_u_) where **G** is the genomic relationship matrix (GRM) as described in VanRaden [37] (method 2). Finally, $e_{ijkl}$ was the corresponding residual term assumed to be distributed as **e** ~ N(0,σ^2^_e_).

The heritability was calculated as:

$$h^{2} = \frac{{\sigma_{a}^{2} }}{{\sigma_{a}^{2} + \sigma_{e}^{2} }}$$

where $\sigma_{a}^{2}$ is the additive variance and $\sigma_{e}^{2}$ is the residual variance.

A total of 300,000 Gibbs sampling iteration were drawn with a burn-in of 100,000 and a thin interval of 10. The posterior mean was represented using the viz_forest function [38]. The uncertainty about the heritability (h^2^) estimates was calculated using a confidence interval of a 95% High Posterior Density (HPD) [39].

## ARG-sharing networks

ARG networks were constructed using the output file from the SqueezeMeta pipeline which contains the information about the taxonomy. We selected the 25 most prevalent ARGs cited above filtering by their antibiotic resistance ontology (ARO). Superior Eukaryotes (Table 3) were removed from the taxonomy.

Next, a squared matrix was created, containing the number of times an ARG is shared between two phyla. For the ARG to be considered, there must be a coincidence between the two phyla in each herd independently. Obtained values are codified to show the strength of the relationship, assigning 1 if the mean of the coincidences is within the interval (1.98,7.41], 2 for (7.41,12.8], 3 for (12.8,18.2] and 4 for (18.2,23.7]. Then, nodes were represented using Cytoscape [40]. Phyla were considered as the nodes, and the attributes (colour of the lines) were the number of ARGs shared. A shape was assigned to each phylum according to the kingdom they belong to and a different colour to the lines that represents the interactions according to their strength, which is given by the amount of ARGs shared.

## Data Availability

The data that support the findings of this study are available from the ENA repository accessible at https://www.ncbi.nlm.nih.gov/bioproject/PRJEB44278.

## Abbreviations

ARG: Antibiotic resistance gene
RA: Relative abundance
CoDa: Compositional data
CARD: Comprehensive antibiotic resistance database
HGT: Horizontal gene transfer
AMR: Antimicrobial resistance
ARO: Antibiotic resistance ontology

## References

[1] Taylor LH, Latham SM, Woolhouse MEJ (2001). Risk factors for human disease emergence. Philos Trans R Soc B Biol Sci R Soc.

[2] Shears P (2000). Antimicrobial resistance in the tropics. Trop Doct.

[3] European Union. Ban on antibiotics as growth promoters in animal feed enters into effect. Regulation (EC) No 1831/2003 on additives for use in animal nutrition. 2006;1.

[4] Penders J, Stobberingh EE, Savelkoul PHM, Wolffs PFG (2013) The human microbiome as a reservoir of antimicrobial resistance. Front Microbiol 4:1–7. 10.3389/fmicb.2013.00087 PMC3627978 23616784

[5] Hoque MN, Istiaq A, Clement RA, Gibson KM, Saha O, Islam OK, et al. Insights into the resistome of bovine clinical mastitis microbiome, a key factor in disease complication. Front Microbiol Front. 2020;11:860. 10.3389/fmicb.2020.00860 PMC7283587 32582039

[6] Bradley AJ (2002). Bovine mastitis: an evolving disease. Vet J.

[7] Zhang Y, Zhang C, Parker DB, Snow DD, Zhou Z, Li X (2013). Occurrence of antimicrobials and antimicrobial resistance genes in beef cattle storage ponds and swine treatment lagoons. Sci Total Environ.

[8] Mathew AG, Cissell R, Liamthong S. Antibiotic resistance in bacteria associated with food animals: a United States perspective of livestock production. Foodborne Pathog. Dis. 2007. 4:115–133. 10.1089/fpd.2006.0066 17600481

[9] Price LB, Graham JP, Lackey LG, Roess A, Vailes R, Silbergeld E. Elevated risk of carrying gentamicin-resistant escherichia coli among U.S. poultry workers. Environ Health Perspect. 2007;115:1738–42. 10.1289/ehp.10191 PMC2137113 18087592

[10] Van Den Bogaard AE, Stobberingh EE (2000). Epidemiology of resistance to antibiotics: links between animals and humans. Int J Antimicrob Agents.

[11] Gonzalez-Recio O, Zubiria I, García-Rodríguez A, Hurtado A, Atxaerandio R (2018). Short communication: Signs of host genetic regulation in the microbiome composition in 2 dairy breeds: Holstein and Brown Swiss. J Dairy Sci.

[12] Pyörälä S, Baptiste KE, Catry B, van Duijkeren E, Greko C, Moreno MA, et al. Macrolides and lincosamides in cattle and pigs: use and development of antimicrobial resistance. Vet J. 2014; 230–239. 10.1016/j.tvjl.2014.02.028 24685099

[13] Pyörälä S, Taponen J, Katila T (2014). Use of antimicrobials in the treatment of reproductive diseases in cattle and horses. Reprod Domest Anim.

[14] Granja RHMM, Nino AMM, Reche KVG, Giannotti FM, de Lima AC, Wanschel ACBA (2013). Determination and confirmation of metronidazole, dimetridazole, ronidazole and their metabolites in bovine muscle by LC-MS/MS. Food Addit Contam Part A Chem Anal Control Expo Risk Assess.

[15] Amundson Romich, J. (2011). Fundamentals of pharmacology for veterinary technicians, 2nd edn. Cengage.

[16] Lin L, Huang X, Yang H, He Y, He X, Huang J (2021). Molecular epidemiology, antimicrobial activity, and virulence gene clustering of *Streptococcus agalactiae* isolated from dairy cattle with mastitis in China. J Dairy Sci.

[17] EMEA, Veterinary Medicines and Inspections. Committee for medicinal products for veterinary use phenoxymethypenicillin. 2005;1–3.

[18] Constance Mackworth-Young, Rudo Chingono, Constancia Mavodza, Grace McHugh, Mandikudza Tembo, Chido Dziva Chikwari, Helen A Weiss, Simbarashe Rusakaniko, Sithembile Ruzario SB& RF. Bull World Health Org. 2021. 10.2471/BLT.20.260224 PMC7856363 33551502

[19] Crofts TS, Gasparrini AJ, Dantas G. Next-generation approaches to understand and combat the antibiotic resistome. Nat Rev Microbiol 2017;422–34. 10.1038/nrmicro.2017.28 PMC5681478 28392565

[20] Li RW, Connor EE, Li C, Baldwin Vi RL, Sparks ME (2012). Characterization of the rumen microbiota of pre-ruminant calves using metagenomic tools. Environ Microbiol.

[21] Auffret MD, Dewhurst RJ, Duthie CA, Rooke JA, John Wallace R, Freeman TC, et al. The rumen microbiome as a reservoir of antimicrobial resistance and pathogenicity genes is directly affected by diet in beef cattle. Microbiome. 2017;5:159. 10.1186/s40168-017-0378-z. 10.1186/s40168-017-0378-z PMC5725880 29228991

[22] Xue M-Y, Xie Y-Y, Zhong Y-F, Liu J-X, Guan LL, Sun H-Z. Ruminal resistome of dairy cattle is individualized and the resistotypes are associated with milking traits. Anim Microbiome 2021 31.; 2021;3:1–17. 10.1186/s42523-021-00081-9 PMC7877042 33568223

[23] Ofria C, Adami C, Collier TC (2003). Selective pressures on genomes in molecular evolution. J Theor Biol.

[24] Wijesekara PNK, Kumbukgolla WW, Jayaweera JAAS, Rawat D. Review on usage of vancomycin in livestock and humans: maintaining its efficacy, prevention of resistance and alternative therapy. Vet Sci. 2017;4(1):6. 10.3390/vetsci4010006. 10.3390/vetsci4010006 PMC5606620 29056665

[25] Sun H-Z, Peng K-L, Xue M-Y, Liu J-X. Metagenomics analysis revealed the distinctive ruminal microbiome and resistive profiles in dairy buffaloes. Anim Microb 2021;3:1–13. 10.1186/s42523-021-00103-6 PMC8247143 34210366

[26] Andrade-Martínez JS, Moreno-Gallego JL, Reyes A (2019). Defining a core genome for the herpesvirales and exploring their evolutionary relationship with the caudovirales. Sci Rep.

[27] McKenna M (2013). Antibiotic resistance: the last resort. Nature.

[28] VMD. UK Veterinary Antibiotic Resistance and Sales Surveillance (VARSS) 2012. VMD. 2013.

[29] Deusch S, Camarinha-Silva A, Conrad J, Beifuss U, Rodehutscord M, Seifert J (2017). A structural and functional elucidation of the rumen microbiome influenced by various diets and microenvironments. Front Microbiol..

[30] Saborío-Montero A, Gutiérrez-Rivas M, García-Rodríguez A, Atxaerandio R, Goiri I, López de Maturana E, et al. Structural equation models to disentangle the biological relationship between microbiota and complex traits: methane production in dairy cattle as a case of study. J Anim Breed Genet 2020;137:36–48. 10.1111/jbg.12444 31617268

[31] Browning BL, Zhou Y, Browning SR (2018). A one-penny imputed genome from next-generation reference panels. Am J Hum Genet.

[32] Tamames J, Puente-Sánchez F. SqueezeMeta, a highly portable, fully automatic metagenomic analysis pipeline. Front Microbiol. 2019;10:3349. 10.3389/fmicb.2018.03349 PMC6353838 30733714

[33] McArthur AG, Waglechner N, Nizam F, Yan A, Azad MA, Baylay AJ (2013). The comprehensive antibiotic resistance database. Antimicrob Agents Chemother.

[34] Martín-Fernández JA, Hron K, Templ M, Filzmoser P, Palarea-Albaladejo J (2015). Bayesian-multiplicative treatment of count zeros in compositional data sets. Stat Model.

[35] Palarea-Albaladejo J, Martín-Fernández JA (2015). ZCompositions - R package for multivariate imputation of left-censored data under a compositional approach. Chemom Intell Lab Syst.

[36] Legarra A, Varona L, López de Maturana E. Threshold Model 1–33. 2011. http://genoweb.toulouse.inra.fr/~alegarra/tm_folder/manualtm.pdf.

[37] VanRaden PM (2008). Efficient methods to compute genomic predictions. J Dairy Sci J Dairy Sci.

[38] Kossmeier M, Tran US, Voracek M. Metaviz: forest plots, funnel plots, and visual funnel plot inference for meta-analysis [R package metaviz version 0.3.1]. 2020.

[39] Lewis S, Clarke M (2001). Forest plots: trying to see the wood and the trees. Br Med J.

[40] Shannon P, Markiel A, Ozier O, Baliga NS, Wang JT, Ramage D (2003). Cytoscape: a software environment for integrated models of biomolecular interaction networks. Genome Res.

